# Ginseng Berry Extract Supplementation Improves Age-Related Decline of Insulin Signaling in Mice

**DOI:** 10.3390/nu7043038

**Published:** 2015-04-22

**Authors:** Eunhui Seo, Sunmi Kim, Sang Jun Lee, Byung-Chul Oh, Hee-Sook Jun

**Affiliations:** 1 College of Pharmacy and Gachon Institute of Pharmaceutical Science, Gachon University, Incheon 406-840, Korea; E-Mail: eunhuiseo@gachon.ac.kr; 2 Lee Gil Ya Cancer and Diabetes Institute, Gachon University, Incheon 406-840, Korea; E-Mail: bcoh@gachon.ac.kr; 3 R & D Center, Amorepacific Corporation, Gyeonggi-do 446-729, Korea; E-Mails: apsun20@amorepacific.com (S.K.); leesjun2006@gmail.com (S.J.L.); 4 Gachon Medical Research Institute, Gil Hospital, Incheon 405-760, Korea

**Keywords:** ginseng berry, aging, insulin sensitivity

## Abstract

The aim of this study was to evaluate the effects of ginseng berry extract on insulin sensitivity and associated molecular mechanisms in aged mice. C57BL/6 mice (15 months old) were maintained on a regular diet (CON) or a regular diet supplemented with 0.05% ginseng berry extract (GBD) for 24 or 32 weeks. GBD-fed mice showed significantly lower serum insulin levels (*p* = 0.016) and insulin resistance scores (HOMA-IR) (*p* = 0.012), suggesting that GBD improved insulin sensitivity. Pancreatic islet hypertrophy was also ameliorated in GBD-fed mice (*p* = 0.007). Protein levels of tyrosine phosphorylated insulin receptor substrate (IRS)-1 (*p* = 0.047), and protein kinase B (AKT) (*p* = 0.037), were up-regulated in the muscle of insulin-injected GBD-fed mice compared with CON-fed mice. The expressions of forkhead box protein O1 (FOXO1) (*p* = 0.036) and peroxisome proliferator-activated receptor gamma (PPARγ) (*p* = 0.032), which are known as aging- and insulin resistance-related genes, were also increased in the muscle of GBD-fed mice. We conclude that ginseng berry extract consumption might increase activation of IRS-1 and AKT, contributing to the improvement of insulin sensitivity in aged mice.

## 1. Introduction

Aging is an inescapable procedure caused by the interaction of biological and psychosocial factors [1]. It is well known that aging is associated with a decline in insulin action. Insulin resistance results from impairment of the insulin signaling pathway, but the molecular mechanisms are unclear. Insulin resistance is related to several well-known age-related changes, such as increased adiposity, decreased muscle mass, mitochondrial dysfunction, hormonal changes, increased oxidative stress and inflammation, changes in dietary habits and reduced physical activity [2]. Insulin resistance is also one of the characteristics of metabolic syndrome and the pre-diabetic state [3]. The pancreatic beta cell mass changes according to insulin demand, and the condition of insulin resistance requires higher levels of insulin. Thus, pancreatic islets adapt to insulin resistance through a complex set of changes, including beta cell hyperplasia and hypertrophy [4].

There is a growing interest in natural product-based dietary supplements to counteract aging-related disease. Ginseng (*Panax* genus) has been used as a traditional medicine for thousands of years in Korea and Asia. Ginseng has pharmacological properties, including anti-cancer [5,6], anti-aging [7] and anti-allergic effects [8,9]. Ginseng has also received increasing attention as a complementary and alternative medicine for the treatment of diabetes. Ginseng extract treatment has been reported to have hypoglycemic effects in animal models of types 1 and 2 diabetes [10,11,12]. A previous study demonstrated that ginseng berry (GB) extract showed better anti-hyperglycemic activity than ginseng root extract when used at the same concentration [13], and the consumption of GB extract increased insulin secretion and ameliorated hyperglycemia in diabetic mice [14]. However, the effects of GB extract on insulin sensitivity in the aged condition have not been specifically examined. In this study, we investigated the effects of the consumption of GB extract on glucose levels and insulin sensitivity in peripheral tissues in aged mice.

## 2. Materials and Methods

### 2.1. Materials

Dulbecco’s Modified Eagle’s Medium (DMEM), Ham’s F12 medium, ITS™ premix and fetal bovine serum (FBS) were purchased from Gibco BRL (Grand Island, NY, USA). Antibodies against protein kinase B (AKT), phosphorylated protein kinase B (pAKT), insulin receptor substrate (IRS)-1 and phospho-IRS-1 (Ser307) were obtained from Cell Signaling (Boston, MA, USA). Antibody against phospho-tyrosine (4G10) was obtained from Millipore (Bilerica, MA, USA). Horseradish peroxidase-conjugated secondary antibodies were obtained from Santa Cruz Biotechnology Inc. (Santa Cruz, CA, USA). Anti-glucagon and anti-insulin antibodies were obtained from Dako (Carpinteria, CA, USA).

### 2.2. Preparation of Korean GB Extract

Korean GB (*Panax ginseng*, C. A. Meyer) were harvested, and the seeds were separated and removed. Next, the pulp and juice were dried in hot air and refluxed with 70% ethanol for 10 h. The extract was filtered and concentrated under reduced pressure at 45 °C to obtain standardized Korean GB extract.

### 2.3. Cell Culture

Mouse skeletal muscle cells (C2C12 cell line) were obtained from the American Type Culture Collection (ATCC, Rockville, MD, USA). Cells were grown at 37 °C and 5% CO_2_ in a humidified chamber. C2C12 myoblast cells were cultured in growing medium (Dulbecco’s modified Eagles medium (DMEM) supplemented with 10% fetal bovine serum (FBS) and 1% penicillin-streptomycin solution). Myogenic differentiation was induced on confluent cultured cells by changing the growth medium to differentiation medium (DMEM supplemented with 2% horse serum instead of FBS). The differentiation medium was replaced daily. C2C12 cells were differentiated for 1 week and then treated with 100 μM H_2_O_2_ with or without GB extract (10 or 100 μg mL^−1^) for 24 h. After 2 min of 50 μM insulin treatment, cells were harvested for western blot analysis.

### 2.4. Animals

15-month-old male C57BL/6 mice were supplied by the Korea Research Institute of Bioscience and Biotechnology (Daejeon, Korea). Animals were maintained in the animal facilities at the Lee Gil Ya Cancer and Diabetes Institute, Gachon University of Medicine and Science, under a 12 h light, 12 h dark photoperiod. All animal experiments were carried out under a protocol approved by the Institutional Animal Care and Use Committee at Lee Gil Ya Cancer and Diabetes Institute, Gachon University. After adaptation for one week, mice were provided with either a regular diet (AIN-93G, Research diets Inc., NJ, USA; CON) or a diet containing 0.05% GB extract by weight added to AIN-93G (GBD), for 6 months (24 weeks) or 8 months (32 weeks).

### 2.5. Glucose Tolerance Tests

After 22 weeks of GBD consumption, animals were fasted overnight and glucose (2 g kg^−1^ body weight) was administered by intraperitoneal injection. Blood samples were obtained from the tail vein at 0, 30, 60, 90 and 120 min after glucose load. Blood glucose levels were measured with a glucose analyzer (OneTouch^®^ Ultra, Lifescan, Johnson & Johnson, Milpitas, CA, USA).

### 2.6. Insulin Tolerance Tests

After 22 weeks of GBD consumption, animals were fasted for 4 h and insulin (0.75 U kg^−1^ body weight; Humilin; Lilly, Indianapolis, IN, USA) was administered by intraperitoneal injection. Blood glucose was measured at 0, 30, 60 and 90 min after injection.

### 2.7. Measurement of Blood Glucose, Serum Insulin and Serum Lipid Level

Blood samples were collected before sacrifice to measure blood glucose and serum insulin. The blood sampling line was filled with a solution of 4.5% ethylenediaminetetraacetic acid (EDTA) to prevent blood clotting. Samples were kept on ice, and serum was isolated and stored at −70 °C until analysis. Glucose levels were measured with a glucose analyzer (OneTouch^®^ Ultra). Insulin levels were determined in duplicate using 5 μL of serum and an UltraSensitive Mouse Insulin kit (ALPCO, Windham, NH, USA) according to the manufacturer’s instructions. Serum levels of alanine cholesterol, triglycerides, low-density lipoprotein (LDL)-cholesterol and high-density lipoprotein (HDL)-cholesterol were measured using Beckman Coulter AU680 chemistry analyzer (Beckman Coulter, Inc., Brea, CA, USA).

### 2.8. Measurement of Fat Mass

Fat and lean body masses were assessed by a ^1^H minispec system (Bruker BioSpin) after 24 weeks of GBD feeding.

### 2.9. HOMA-IR Calculation

Animals were fasted for 16 h, blood samples were collected for measurement of blood glucose levels and serum insulin levels were measured using a mouse insulin Enzyme-Linked Immunosorbent Assay (ELISA) kit (ALPCO). An insulin resistance index, the Homeostatic Model Assessment for Insulin Resistance (HOMA-IR), was computed using the following formula:

HOMA-IR = fasting insulin (μIU mL^−1^) × fasting glucose (mmol mL^−1^)/22.5

### 2.10. Immunohistochemical and Histological Staining of Pancreatic Sections

Mice were killed and the pancreata were removed, fixed in 10% formalin, embedded in paraffin and sectioned. Pancreatic sections were deparaffinized in xylene, dehydrated in alcohol, and washed in water. After antigen unmasking, the slides were permeabilized in 0.5% Triton X-100, and non-specific protein binding sites were saturated with 2% bovine serum albumin in PBS for 1 h. The tissue sections were incubated with primary antibodies (rabbit anti-insulin, 1:100; mouse anti-glucagon, 1:100) overnight in a cold room, washed and incubated with rhodamine-conjugated anti-rabbit and fluorescein isothiocyanate-conjugated anti-mouse secondary antibodies for 30 min. Nuclei were then fluorescently labeled with 4’,6-diamidino-2-phenylindole (DAPI). The labeled cells were observed under a confocal microscope (LSM 700, Carl Zeiss Inc., Oberkochen, West Germany).

### 2.11. Western Blotting and Immunoprecipitation

Tissues or cells were homogenized with Mammalian Protein Extraction Buffer (GE Healthcare, Milwaukee, WI, USA) containing a protease and phosphatase inhibitor cocktail (Sigma-Aldrich). For immunoprecipitation, the lysates were centrifuged to remove the insoluble material, and supernatants were precleared with protein A-Sepharose beads for 15 min at 4 °C. Thereafter, supernatants were incubated with IRS-1 antibodies for 16 h at 4 °C. Protein A-Sepharose was added to the reaction mixture and the incubation was continued for two additional hours. Beads were collected by centrifugation and washed eight times with cold lysis buffer, resuspended in Laemmli sample buffer, and boiled for 3 min. The immunoprecipitate or total proteins (30–50 μg) were resolved by 8% or 15% sodium dodecyl sulfate polyacrylamide gel electrophoresis, transferred onto membranes, and blocked with tris buffered saline containing Tween 20% in 5% non-fat dry milk. The membranes were incubated with specific primary antibodies and visualized by incubating with horseradish peroxidase-conjugated secondary antibodies followed by Immobilon Western Chemiluminescent HRP Substrate (Millipore, St. Charles, MO, USA). Chemiluminescence was detected by LAS-4000 (Fuji Film, Tokyo, Japan). The images derived from western blotting were analyzed through ImageJ (National Institutes of Health, Bethesda, MD, USA) software for Windows.

### 2.12. Quantitative Real-Time-PCR (qRT-PCR) Analysis

The total RNA was extracted from the cultured cells using TRIZOL reagent (Invitrogen Corp., Carlsbad, CA, USA) following the manufacturer’s instructions, and cDNA was synthesized using a PrimeScript 1st strand cDNA synthesis kit (Takara Bio Inc., Kyoto, Japan). qRT-PCR was performed using the SYBR Premix Ex Taq II, ROX plus (Takara Bio Inc., Kyoto, Japan) and the Prism 7900HT sequence detection system (Applied Biosystems, Foster City, CA, USA). PCR was carried out for 40 cycles (2 min at 50°, 10 min at 95°, and 40 cycles of 10 s at 95° and 1 min at 60°). The relative copy number was calculated using the threshold crossing point (Ct) as calculated by ΔΔCt. Primer sequences were as follows: 5′–TGGAGAGCACCAAGACAGACA–3′ and 5′–TGCCGGAGTCGACAATGAT–3′ for mouse cyclophlin; 5′–TCATGGATGGAGATACCTTGGA–3′ and 5′–CTTGACACTGTGTGGGAAGCTT–3′ for mouse forkhead box (FOX) O1; and 5′–ACAGGAGAATCTCCCAGAGTTTC–3′ and 5′–CACCAGTGTGAATTACAGCAAATC–3′ for mouse peroxisome proliferator-activated receptor γ (PPAR γ).

### 2.13. Statistical Analyses

All data are expressed as mean ± standard error of at least three independent experiments. Data were analyzed by one-way or two-way ANOVA, followed by *post hoc* tests (SPSS 10.0 statistical software). *p*-Values less than 0.05 were considered statistically significant.

## 3. Results

### 3.1. GB Extract Supplementation Did not Affect the Body Weight, Blood Glucose Levels or Serum Lipid Levels in Aged Mice

**Figure 1:**
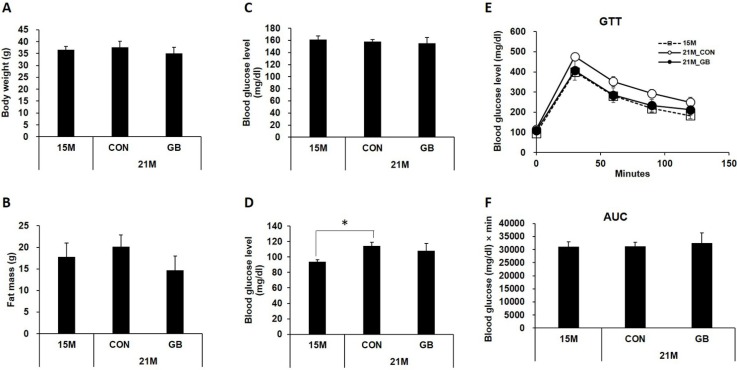
Ginseng berry (GB) extract supplementation does not change the body weight or blood glucose in aged mice. Fifteen-month-old mice were fed a regular diet (CON) or a regular diet supplemented with 0.05% GB extract for 6 months. At 21 months of age, (**A**) body weights were measured; (**B**) whole body fat mass was measured by a ^1^H minispec system (LF-90II, Bruker Optics, Germany); and (**C**) non-fasting and (**D**) fasting blood glucose levels were measured; (**E**) glucose tolerance tests were performed as well and (**F**) the area under the curve (AUC) was calculated. Values are means ± SE, *n* = 8 group^−1^.

**Figure 2:**
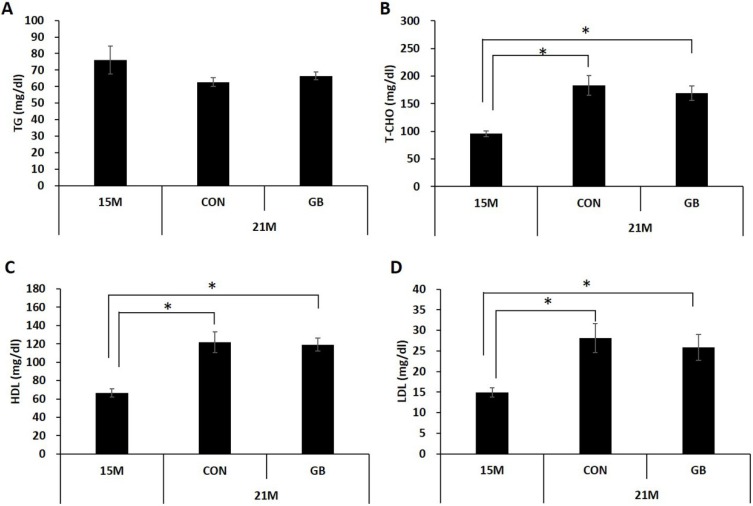
Ginseng berry (GB) extract supplementation does not change plasma lipid profile in aged mice. Fifteen-month-old mice were fed a regular diet (CON) or a regular diet supplemented with 0.05% GB extract for 6 months. (**A**) Triglyceride (TG); (**B**) total cholesterol (T-CHO); (**C**) HDL-cholesterol and (**D**) LDL-cholesterol level in the plasma were measured at 21 months of age. Values are mean ± SE, (*n* = 8 group^−1^), * *p*< 0.05.

To examine the effects of GB extract on metabolic parameters in aged mice, 15-month-old C57BL/6 mice were fed with a diet supplemented with 0.05% GB extract (GBD) or a regular diet (CON) for 24 weeks and body weight, fat mass, and blood glucose levels were examined. There were no significant differences in body weight (Figure 1A) or fat mass (Figure 1B) between GBD-fed and CON-fed mice at 21 months of age. No difference was observed in non-fasting (Figure 1C) or fasting (Figure 1D) blood glucose levels between GBD-fed group and CON-fed group. Blood glucose levels were normal range in all groups. Glucose tolerance tests showed that the ability to clear exogenous glucose was not significantly different between GBD-fed and CON-fed mice (Figure 1E,F). There was no change in plasma triglyceride levels associated with aging, and GBD feeding did not change plasma triglyceride levels in 21-month-old mice (Figure 2A). While plasma cholesterol (total, HDL and LDL cholesterol) levels were increased in 21-month-old mice compared to 15-month-old mice, GBD feeding did not change these levels compared with CON-fed mice (Figure 2B–D).

### 3.2. GB Extract Supplementation Increased Insulin Sensitivity in Aged Mice

**Figure 3:**
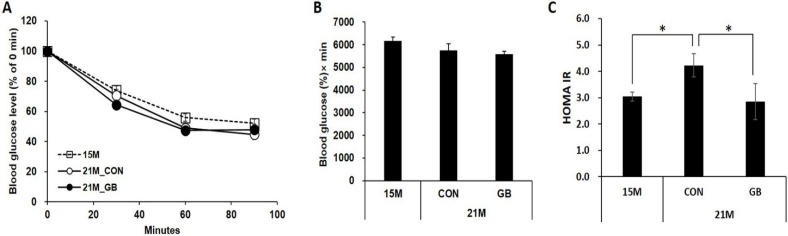
Ginseng berry (GB) extract supplementation improves insulin sensitivity in aged mice. Fifteen-month-old mice were fed a regular diet (CON) or a regular diet supplemented with 0.05% GB extract for 24 weeks. Insulin was administered and (**A**) blood glucose levels and (**B**) the area under the blood glucose curve (AUC) were measured at 21 months of age; (**C**) The insulin resistance scores (HOMA-IR) score was calculated. Values are means ± SE, *n* = 8 group^−1^, * *p* < 0.05.

To determine whether GB extract supplementation affects insulin sensitivity, we performed insulin tolerance tests after 22 weeks of GBD feeding. Insulin tolerance tests showed no difference in blood glucose levels at the tested time points between GBD-fed and CON-fed groups (Figure 3A,B). Therefore, we analyzed insulin sensitivity by calculating the HOMA-IR. The HOMA-IR was significantly elevated at 21 months of age compared with 15 months of age (3.0 ± 0.7 and 4.2 ± 0.7, respectively; *p* = 0.026) indicating that insulin sensitivity was reduced during aging. GB extract supplementation significantly reduced the HOMA-IR score to 2.9 ± 0.7 (Figure 3C).

### 3.3. GB Extract Supplementation Ameliorated Pancreatic Islet Hypertrophy in Aged Mice and Decreased Serum Insulin Levels

**Figure 4:**
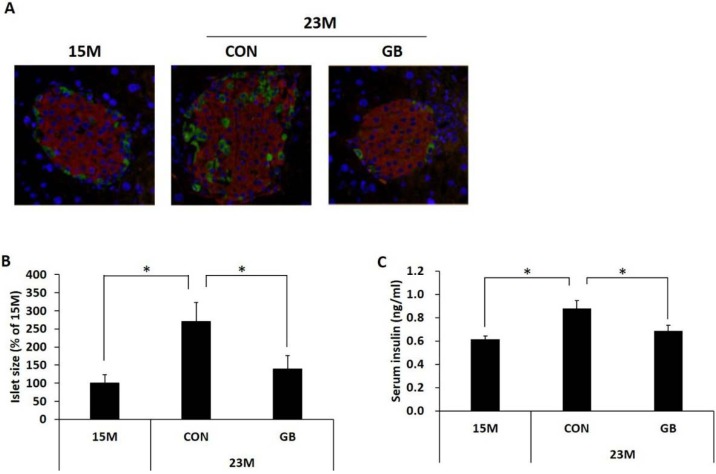
Ginseng berry (GB) extract supplementation decreases serum insulin levels and ameliorates pancreatic islet hypertrophy in aged mice. Fifteen-month-old mice were fed a regular diet (CON) or regular diet supplemented with 0.05% ginseng berry extract (GBD) for 8 months. At 23 months of age, the pancreas was removed and (**A**) pancreatic sections were stained with anti-insulin (red) and anti-glucagon (green) antibodies. Nuclei were fluorescently labeled with 4’,6-diamidino-2-phenylindole (DAPI) (blue); (**B**) Islet size was measured at 23 months of age (≥25 islets were measured from each mouse, *n* = 5 group^−1^); (**C**) Non-fasting serum insulin level was measured at 23 months of age. Values are means ± SE. * *p* < 0.05.

To investigate the effect of GB extract supplementation on pancreatic islets, we first examined islet morphology by immunohistochemistry. The pancreatic islets of the 23-month-old mice were hypertrophic, with islet size increased by approximately 270%, and there was increased immunostaining with antibodies against insulin compared with 15-month-old mice. The arrangement of alpha cells and beta cells in pancreatic islets of 23-month-old mice was also disordered compared with 15-month-old mice. However, GBD feeding prevented the enlargement and disturbance of the islets (Figure 4A,B). Non-fasting serum insulin levels were increased at 23 months of age compared with 15 month of age, and GBD feeding significantly lowered the serum insulin levels (Figure 4C).

### 3.4. GB Extract Supplementation Increased Phosphorylation of IRS-1 and AKT in Muscle of Aged Mice

**Figure 5:**
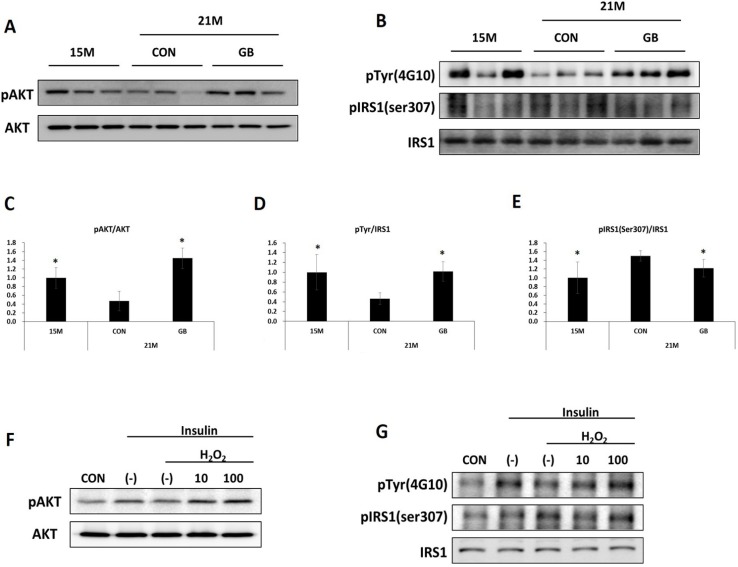

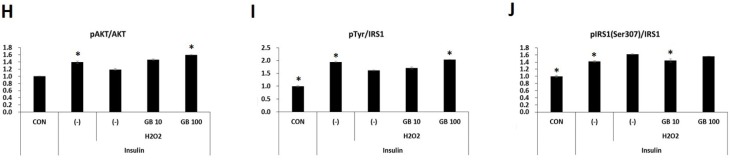
Ginseng berry (GB) extract supplementation increases protein kinase B (AKT) and insulin receptor substrate-1 (IRS-1) in skeletal muscle of aged mice. (**A**–**E**) Fifteen-month-old mice were fed a regular diet (CON) or regular diet supplemented with 0.05% ginseng berry extract (GBD) for 6 months (*n* = 8 in each group). Skeletal muscle tissue was collected 7 min after insulin injection (2 U kg^−1^, i.p.). (**A**) Protein expression of AKT and its phosphorylated form (pAKT) was determined by western blot. (**B**) IRS-1 was immunoprecipitated from whole lysate of skeletal muscle with anti-IRS-1 antibody, and immunoprecipitates were immunoblotted with anti-IRS-1, anti-tyrosine (anti-Tyr) or anti-phospho IRS-1 (anti-pIRS-1) (Ser307) antibodies. (**C**–**E**) The images from (**A**) and (**B**) were analyzed through ImageJ software (National Institutes of Health, Bethesda, MD, USA) for Windows. * *p* < 0.05 *vs.* 21 months CON. (**F**–**J**) C2C12 cells were differentiated for 1 week and then treated with 100 μM H_2_O_2_ with or without GB (10 or 100 μg mL^−1^) for 24 h. After 2 min of 50 μM insulin treatment, cells were harvested. (**F**) Protein expression of AKT and pAKT was determined. (**G**) IRS-1 was immunoprecipitated from whole cell lysate with anti-IRS-1 antibody, and immunoprecipitates were immunoblotted with anti-IRS-1, anti-phospho- tyrosine (anti-p-Tyr) or anti-pIRS-1 (Ser307) antibodies. (**H**–**J**) The images from (**F**) and (**G**) were analyzed through ImageJ software for Windows. * *p* < 0.05 *vs.* insulin treated H_2_O_2_-(–).

As insulin sensitivity seemed to be increased by GBD feeding, we analyzed the effect of GB extract supplementation on insulin signaling molecules in skeletal muscle. AKT is implicated in the insulin-signaling pathway and facilitates glucose uptake in liver and muscle [15]. Thus, phosphorylated and total levels of AKT were analyzed in skeletal muscle by western blot analysis. GBD feeding significantly prevented the reduction of phosphorylated protein kinase B (p-AKT) seen after insulin injection in aged mice (Figure 5A,C). There was no difference in the total levels of AKT. IRS-1 plays a crucial role in determining insulin resistance. It is well known that impaired insulin signal transduction caused by the inhibition of tyrosyl phosphorylation of IRS-1 and the enhancement of serine307 phosphorylation of IRS-1 [16]. Total and tyrosine phosphorylated levels of IRS-1 were increased in the GBD-fed group after insulin injection compared with the CON-fed group (Figure 5B,D). In addition, GBD feeding significantly prevented the increase of phosphorylation of serine307 IRS-1 seen after insulin injection in aged mice (Figure 5B,E). These results suggest that GB extract feeding restored insulin signaling, which was reduced during aging.

### 3.5. GB Extract Supplementation Increased Phosphorylation of IRS-1 and AKT in C2C12 Cells

To investigate whether GB extract also improves insulin sensitivity in the C2C12 mouse muscle cell line, we analyzed levels of AKT and IRS-1 after an H_2_O_2_ load, which mimics aging [17,18]. H_2_O_2_ treatment decreased the phosphorylation of AKT that was induced by insulin treatment, but GB extract supplementation recovered AKT phosphorylation in a dose-dependent manner (Figure 5F,H). Similarly, H_2_O_2_ treatment decreased tyrosyl phosphorylation of IRS-1 that was induced by insulin treatment, but GB extract supplementation recovered tyrosyl phosphorylation in a dose-dependent manner (Figure 5G,I). H_2_O_2_ treatment elevated IRS-1 serine307 phosphorylation in C2C12 after insulin treatment, but GB extract supplementation significantly reduced this to normal levels (Figure 5G,J).

### 3.6. GB Extract Supplementation Increased the Expression of FOXO1 and PPARγ mRNA and Protein

**Figure 6:**
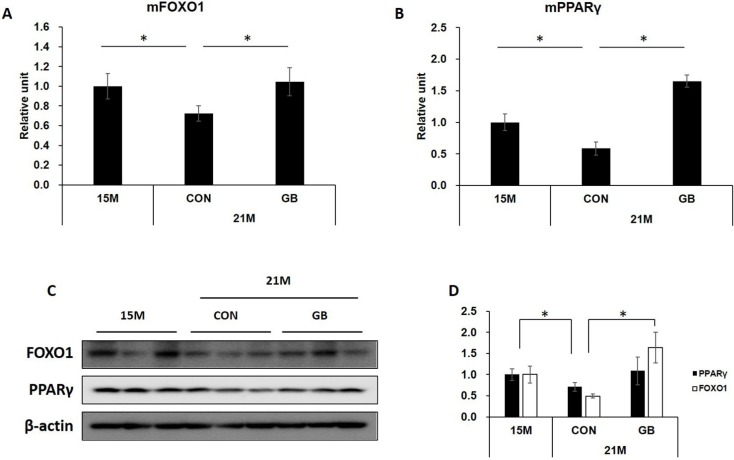
Ginseng berry (GB) extract supplementation increases the expression of forkhead box protein O1 (FOXO1) and peroxisome proliferator activated receptor gamma (PPARγ) in skeletal muscle of aged mice. Fifteen-month-old mice were fed a regular diet (CON) or regular diet supplemented with 0.05% ginseng berry extract (GBD) for 6 months (*n* = 8 in each group). Skeletal muscle tissues were collected 7 min after insulin injection (2 U kg^−1^, i.p.). (**A**) FOXO1 and (**B**) PPARγ mRNA levels were measured by qRT-PCR. Relative units were determined as a ratio of mRNA levels of 15-month-old mice; (**C**) Protein expression of FOXO1 and PPARγ was determined by western blot; (**D**) The images derived from (**C**) were analyzed through ImageJ software for Windows. Values are means ± SE. * *p* < 0.05.

Insulin responses are decreased in skeletal muscle in aged condition and FOXO1 and PPARγ are not only well known insulin singling related genes [19,20], but also are aging-related genes [21,22]. Thus, we investigated whether GB extract supplementation affects the expression of these molecules in skeletal muscle tissues of insulin-injected mice. The expressions of FOXO1 and PPARγ were significantly reduced in 21-month-old mice compared with 15-month-old mice, but GBD feeding induced the recovery of FOXO1 and PPARγ expression levels of both mRNA (Figure 6A,B) and protein (Figure 6C,D). These results demonstrate that GB extract may not only improve insulin signaling, but also may have anti-aging effects.

## 4. Discussion

Metabolic disorders including type 2 diabetes significantly increase with age [23,24]. It is well documented that aging is associated with a decline of insulin action and diminished insulin sensitivity in target tissues, which contributes to the development of age-related glucose intolerance [25,26,27]. In this study, untreated C57BL/6 mice showed markers of increased insulin resistance at 21 months of age compared with 15 months of age, although none of the mice developed full type 2 diabetes. However, GB extract feeding in C57BL/6 mice for 6 months starting at 15 months of age resulted in a significant decrease of HOMA-IR, which is a well-defined biomarker for the assessment of insulin resistance and insulin sensitivity [28,29]. In addition, GB extract consumption significantly decreased fasting insulin levels, although there were no differences in fasting glucose levels, suggesting that the improvement of insulin sensitivity might contribute to the decrease of serum insulin levels.

It is well known that pancreatic beta cells respond to insulin resistance by increasing their cell mass (beta cell hyperplasia) and insulin secretion (hyperinsulinaemia) [30]. Increased insulin secretion is in part related to pancreatic islet hyperplasia and hypertrophy [29,31]. It has been reported that aged mice show significant islet hypertrophy compared with young mice [32,33]. In the present study, hypertrophied islets and increased plasma insulin were observed in 21-month-old mice compared with 15-month-old mice. However, islet hypertrophy and plasma insulin were reduced by GB extract supplementation, which suggests that GB extract feeding reduced insulin resistance and insulin demand, therefore reducing insulin secretion, which depends on the metabolic demand [34].

Insulin resistance in skeletal muscle is a common metabolic disorder in aged individuals and contributes to the development of type 2 diabetes [35]. The insulin signaling pathway is initiated by the binding of insulin to its receptor on cell surfaces and a series of signaling cascades that can be summarized as follows: receptor autophosphorylation and activation of tyrosine kinase, tyrosine phosphorylation of IRS-1 and IRS-2, activation of phosphatidylinositol 3-kinase (PI3K), and activation of AKT and its downstream mediator [36]. Aging is associated with the impairment of IRS-1 function [37]. Many studies have suggested that serine phosphorylation of IRS-1 is elevated in conditions of insulin resistance, and provides negative feedback to insulin signaling to attenuate insulin-stimulated tyrosine phosphorylation [38]. We found that GB extract feeding resulted in an increase of tyrosine phosphorylation and a decrease of serine phosphorylation of IRS-1, leading to improvement of AKT phosphorylation in aged mice. As tyrosine phosphorylation of IRS-1 in the liver and skeletal muscle activates phosphorylation of downstream signaling events, including AKT [15,39], the improvement of AKT phosphorylation by GB extract was the result of regulation of the upstream phosphorylation of the IR/IRS-1/PI3K pathway. Therefore, keeping the normal phosphorylation of IRS-1 may be key to inhibit insulin resistance and improve insulin signaling.

The free-radical theory of aging suggests that many age-related pathologies result from damage to macromolecules by reactive oxygen species [40,41]. Thus, we examined the effect of GB extract on the phosphorylation of IRS-1 subunits after exposure to reactive oxygen species *in vitro*. H_2_O_2_ stimulation increased serine307 phosphorylation and decreased tyrosine phosphorylation of IRS-1 in mouse skeletal muscle cells treated with insulin, similar to the pattern seen in aged mice. As in mice, these changes were prevented by GB extract exposure. These results indicate that GB extract both improved insulin sensitivity and prevented insulin resistance.

FOXO1 and PPARγ are not only well known insulin signaling-related genes [19,20], but also are aging-related genes [21,22]. FOXO1 plays essential roles in the expression of pro-inflammatory mediators and anti-oxidant enzymes [21]. Activation of AKT is the major signaling pathway of insulin action. FOXO1 is also regulated by AKT and has critical roles in the maintenance of skeletal muscle homeostasis. Knockdown of FOXO1 inhibits the protective effect of resveratrol, which is an anti-aging molecule [42]. In this study, we found that aged mice showed a decrease of expression of FOXO1, and GB extract supplementation inhibited this decrease. PPARγ regulates age-related energy metabolism and insulin resistance, and its expression decreases during the aging process [43,44]. Muscle-specific PPARγ-deficient mice develop increased adiposity and whole body insulin resistance [45]. In our study, a decrease of PPARγ expression was observed in skeletal muscle of aged mice, and GB extract supplementation prevented this decrease.

## 5. Conclusions

In conclustion, GB extract feeding inhibits the decrease of expression of FOXO1 and PPARγ, and increases insulin signaling, contributing to the improvement of insulin sensitivity in aged mice.Therefore, GB extract might have a potential to ameliorate age-related metabolic disorder such as diabetes.
